# Tick-transmitted co-infections among erythema migrans patients in a general practice setting in Norway: a clinical and laboratory follow-up study

**DOI:** 10.1186/s12879-021-06755-8

**Published:** 2021-10-08

**Authors:** Knut Eirik Eliassen, Lukas Frans Ocias, Karen A. Krogfelt, Peter Wilhelmsson, Susanne Gjeruldsen Dudman, Åshild Andreassen, Morten Lindbak, Per-Eric Lindgren

**Affiliations:** 1 Department of Global Public Health and Primary Care, University of Bergen, PO Box 7804, 5020 Bergen, Norway; 2 Department of Virus and Microbiological Special Diagnostics, Statens Serum Institut, Artillerivej 5, 2100 Copenhagen, Denmark; 3 Department of Clinical Microbiology, Karlstad Hospital, Region Värmland, 65230 Karlstad, Sweden; 4 Department of Science and Environment, Roskilde University, Universitetsvej 1, 4000 Roskilde, Denmark; 5 Division of Inflammation and Infection, Department of Biomedical and Clinical Sciences, Linköping University, 58185 Linköping, Sweden; 6 Department of Clinical Microbiology, Laboratory Medicine, County Hospital Ryhov, 55185 Jönköping, Sweden; 7 Institute of Clinical Medicine, University of Oslo, 0316 Oslo, Norway; 8 Department of Microbiology, Oslo University Hospital Rikshospitalet, 0424 Oslo, Norway; 9 Department of Virology and Infection Immunology, Norwegian Institute of Public Health, 0213 Oslo, Norway; 10 Faculty of Technology, Natural Sciences and Maritime Technology-INMH, University of South-Eastern Norway-Campus Bø, 3800 Bø, Norway; 11 Antibiotic Centre for Primary Care, Department of General Practice, Institute of Health and Society, University of Oslo, 0316 Oslo, Norway

**Keywords:** Tick-borne infections, General practice, Clinical microbiology, Antibiotic guidelines

## Abstract

**Background:**

Erythema migrans (EM) is the most common manifestation of Lyme borreliosis. Here, we examined EM patients in Norwegian general practice to find the proportion exposed to tick-transmitted microorganisms other than *Borrelia*, and the impact of co-infection on the clinical manifestations and disease duration.

**Methods:**

Skin biopsies from 139/188 EM patients were analyzed using PCR for *Neoehrlichia mikurensis*, *Rickettsia* spp., *Anaplasma phagocytophilum* and *Babesia* spp. Follow-up sera from 135/188 patients were analyzed for spotted fever group (SFG) *Rickettsia, A. phagocytophilum* and *Babesia microti* antibodies, and tested with PCR if positive. Day 0 sera from patients with fever (8/188) or EM duration of ≥ 21 days (69/188) were analyzed, using PCR, for *A. phagocytophilum*, *Rickettsia* spp., *Babesia* spp. and *N*. *mikurensis*. Day 14 sera were tested for TBEV IgG.

**Results:**

We detected no microorganisms in the skin biopsies nor in the sera of patients with fever or prolonged EM duration. Serological signs of exposure against SFG *Rickettsia* and *A*. *phagocytophilum* were detected in 11/135 and 8/135, respectively*.* Three patients exhibited both SFG *Rickettsia* and *A*. *phagocytophilum* antibodies, albeit negative PCR. No antibodies were detected against *B*. *microti*. 2/187 had TBEV antibodies without prior immunization. There was no significant increase in clinical symptoms or disease duration in patients with possible co-infection.

**Conclusions:**

Co-infection with *N*. *mikurensis*, *A*. *phagocytophilum*, SFG *Rickettsia*, *Babesia* spp. and TBEV is uncommon in Norwegian EM patients. Despite detecting antibodies against SFG *Rickettsia* and *A*. *phagocytophilum* in some patients, no clinical implications could be demonstrated.

## Background

Lyme borreliosis (LB) is the most common tick-transmitted infection in the Nordic countries and often presents clinically as a slowly expanding, erythematous skin lesion, known as erythema migrans (EM). It is caused by spirochetes in the *Borrelia burgdorferi* sensu lato (*Bbsl*) group, which are carried and transmitted by *Ixodes ricinus* ticks, the medically most important tick species in northern Europe [1]. Apart from *Bbsl*, *I*. *ricinus* can harbor several other potentially pathogenic microorganisms, including species of spotted fever group (SFG) rickettsiae, *Anaplasma phagocytophilum*, *Neoehrlichia mikurensis*, *Babesia* spp. and tick-borne encephalitis virus (TBEV) [2–7]. Prior studies have demonstrated the concomitant presence of multiple microorganisms in questing ticks and transmission of more than one tick-borne microorganism has been reported in humans [8–11]. Despite this, little attention has been given to the incidence and clinical significance of co-infection with multiple tick-transmitted agents in patients with EM. Furthermore, erythematous skin lesions, similar to those seen in EM, have been associated with or attributed to *N*. *mikurensis* [12–15]. This relationship has, however, never been studied in a larger population sample and no causality has been established. Apart from *N*. *mikurensis*, skin manifestations can be observed in patients infected by other tick-borne microorganisms such as SFG rickettsiae and, less frequently, *A*. *phagocytophilum* [16–18]. As beta-lactam antibiotics, the first-line treatment of patients with EM, are ineffective against most tick-borne microorganisms, it is important to determine to what extent EM skin lesions are co-infected with such microorganisms. Further, it is important to determine the clinical significance of such co-infection and the possibility of skin lesions, similar to EM, being caused by tick-transmitted microorganisms other than *Bbsl*.

In this study, we examined EM-patients from Norway using molecular and serological methods to determine (1) the proportion exposed to tick-transmitted microorganisms other than *Bbsl*, and (2) the impact of co-infection on the clinical manifestations and disease duration of these patients.

## Methods

### Selection of study participants

The study was based on samples collected from 188 patients clinically diagnosed with EM in Norwegian general practice during the years 2012–2013. The EM-patients were originally enrolled in a clinical trial. Thus, the inclusion criteria and study patient characteristics are presented elsewhere [19]. A skin biopsy was taken from 149/188 patients at the time of inclusion (day 0). DNA from *Bbsl* was detected in 104/149 (69.8%) skin biopsies using real-time PCR [19]. Serum samples from all 188 patients were collected at day 0, day 14 and after 3 and 12 months. Although samples were collected as part of a previous study, all analyses and results presented in this paper are new.

### Molecular analyses

**Fig. 1:**
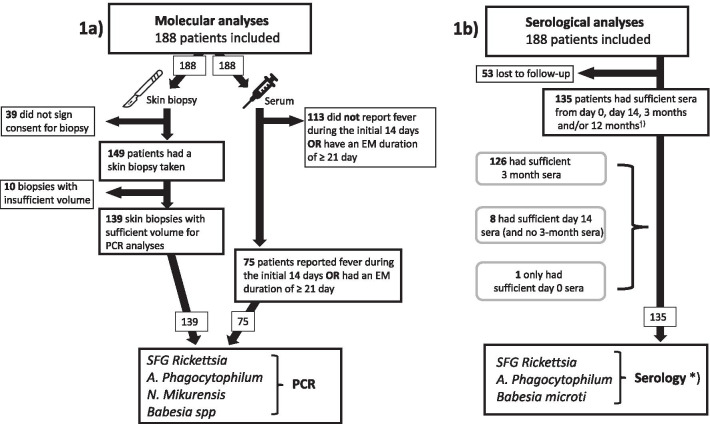
**a** Flowchart describing molecular analyses of EM-patients. **b** Flowchart describing serological analyses of EM-patients. *Day 14 and 3-month sera were additionally analyzed for TBEV (187/188) and *Bbsl* (175/188), respectively. ^†^Patients with detectable IgG in the screening sample also had the remaining study samples tested for antibodies against the corresponding microorganisms

Real-time PCR for tick-borne microorganisms other than *Bbsl* was performed on 139 of the 149 skin biopsies already analyzed for *Bbsl* DNA (Fig. 1a) [19]. Ten of the 149 biopsies contained an insufficient amount of material for further PCR analysis. Real-time PCR (conventional PCR for *Babesia* spp.) was also performed on the inclusion (day 0) serum samples of patients reporting fever during the initial 14 days (8/188) and patients with an EM duration of ≥ 21 days (69/188). This was done as fever is relatively uncommon in patients with EM but is often reported in infections caused by other tick-borne microorganisms. Two patients reported both fever and an EM duration ≥ 21 days.

Most of the molecular analyses were performed at Linköping University, Linköping, SE. A summary of the molecular methods is presented in Table 1a. Total nucleic acids were extracted from the patient specimens and turned into cDNA, as described elsewhere [20]. Different real-time PCR assays were used to detect *N*. *mikurensis*, *Rickettsia* spp., *A*. *phagocytophilum* and *Babesia* spp. Additional PCR analyses were performed on the inclusion (day 0) samples of patients displaying a fourfold or higher rise in antibody titers against SFG *Rickettsia* spp., *A*. *phagocytophilum* or *Babesia microti*. The molecular analyses of sera for *Babesia* spp. was done using conventional PCR at Statens Serum Institut, Copenhagen, DK, using the same primers and target gene as the real-time *Babesia* assay, as described elsewhere [21].

**Table 1:** Overview of the reagents and assays used for the molecular (a) and serological (b) analyses

(a)					
Microorganism	Primer/probe	Nucleotide sequence (5′ → 3′)	Target gene	References	
SFG *Rickettsia* spp.	CS-F	TCGCAAATGTTCACGGTACTTT	gltA	[22]	
	CS-R	TCGTGCATTTCTTTCCATTGTG			
	CS-P	FAM-TGCAATAGCAAGAACCGTAGG CTGGATG-BHQ1			
*A. phagocytophilum*	ApF	TTTTGGGCGCTGAATACGAT	gltA	[6]	
	ApR	TCTCGAGGGAATGATCTAATAACGT			
	ApM	FAM-TGCCTGAACAAGTTATG-BHQ1			
*N*. *mikurensis*	Forward	CGGAAATAACAAAAGATGGA	groEL	[12]	
	Reverse	ACCTCCTCGATTACTTTAG			
	Probe	6FAM-TTGGTGATGGAACTACA-MGB			
*Babesia* spp.	BJ1	GTCTTGTAATTGGAATGATGG	18S rRNA	[24]	
	BN2	TAGTTTATGGTTAGGACTACG			

(b)					
Microorganism	Method	Manufacturer	Antigen	IgG cut-off	IgM cut-off
SFG *Rickettsia* spp.	Indirect IFA	Focus Diagnostics	Inactivated *R*. *rickettsii*	1:64	1:64
*A. phagocytophilum*	Indirect IFA	Focus Diagnostics	Infected HL60 cells	1:64	1:20
*Babesia microti*	Indirect IFA	Focus Diagnostics	Infected erythrocytes	1:64	–
*Bbsl*	Indirect ELISA	Enzygnost (Siemens)	VlsE	^a^	–
TBEV	Indirect ELISA	1. Virion/Serion 2. Euroimmun	1. Inactivated TBEV prepared from strain Moscow B-4 2. Inactivated TBEV prepared from strain K23	1^b^. 2^c^	–

*FAM* 6-carboxy-fluorescine, *BHQ* Black Hole Quencher, *MGB* minor groove binder

^a^According to manufacturer’s instructions

^b^For Virion/Serion cut-offs are calculated for each batch according to manufacturer’s instructions

^c^For Euroimmun there is a fixed negative cut-off of < 120 VIEU/ml

### Real-time PCR assays

#### *Rickettsia* spp.

Detection of *Rickettsia* spp. was done using a TaqMan real-time PCR assay, as previously described [22]. The primers CS-F and CS-R, and probe CS-P are designed to target the *Rickettsia* spp. citrate synthase gene (*gltA*) to amplify a 74-bp long amplicon (Table 1). As a positive control, a synthetic plasmid containing the target sequence of the TaqMan real-time PCR assay was used. The plasmid contained the target sequence, spanning the nucleotides 1102–1231 of the *Rickettsia rickettsii gltA* gene (GenBank: U59729), synthesized and cloned into pUC57 vector (GenScript). The assay has a limit of detection of 10 copies of the plasmid per reaction.

#### Anaplasma phagocytophilum

Detection of *A. phagocytophilum* was done using a TaqMan real-time PCR assay, as previously described [6]. The primers ApF and ApR, and the probe ApM are designed to target the *A. phagocytophilum* citrate synthase gene (*gltA*) to amplify a 64-bp long amplicon (Table 1). As a positive control, a synthetic plasmid containing the target sequence of the TaqMan real-time PCR assay was used. The plasmid contained the target sequence, spanning the nucleotides 304–420 of the *A. phagocytophilum gltA* gene (GenBank: AF304137), synthesized and cloned into pUC57 vector (GenScript, Piscataway, NJ, USA). The assay has a limit of detection of 30 copies of the plasmid per reaction.

#### Neoehrlichia mikurensis

Detection of *N. mikurensis* was done using a TaqMan real-time PCR assay, as previously described [12]. The primers and the probe are designed to target the *N. mikurensis groEL* gene to amplify a 169-bp long amplicon (Table 1). As a positive control, cDNA samples positive for *N. mikurensis* confirmed by sequencing in an earlier study [23] were used in each run.

#### *Babesia* spp.

Detection of *Babesia* spp. in the skin biopsies was done using a SYBR green real-time PCR assay, as previously described [24]. Primers BJ1 and BN2 are designed to target the Babesia *18S* rRNA gene to amplify a 411–452 bp long amplicon (Table 1). As a positive control, a synthetic plasmid containing the target sequence of the SYBR green real-time PCR assay was used. The plasmid contained the target sequence, spanning the nucleotides 467–955 of the *B. divergens 18S* rRNA gene (GenBank: AJ439713), synthesized and cloned into pUC57 vector (GensSript). The assay has a limit of detection of 10 copies of the plasmid per reaction.

### Serological analyses

Samples were analyzed using both indirect immunofluorescence antibody (IFA) assays and ELISA assays as shown in Fig. 1b. A summary of the serological assays used is presented in Table 1b. All samples were examined using current serological “gold standards” with high reported sensitivities and specificities [25–27]. Of the 135 patients who were serologically analyzed for other pathogens than *Bbsl*, 105 (78%) had their skin biopsies undergo molecular analysis using real-time PCR (Table 1a).

### IFA

135 of the 188 patients had sufficient sera for further serological analyses using indirect IFA assays (Fig. 1b). Sufficient 3-month sera were available for 126 of these patients and were screened for the presence of IgG and IgM antibodies against SFG *Rickettsia* and *A*. *phagocytophilum* as well as IgG antibodies against *B. microti*. In patients missing 3-month sera, the day 14 sera (n = 8) and day 0 sera (n = 1) were screened instead (Fig. 1b).

The serological analyses for SFG *Rickettsia*, *A*. *phagocytophilum* and *B. microti* were done at SSI, Copenhagen DK, using commercially available IFA assays (Focus Diagnostics, Inc., Cypress, CA, USA). All samples were analyzed as part of the same experiment, exclusively for the present study. Samples were prepared according to the manufacturer’s instructions and all IFA samples underwent two-fold dilutions until end-point fluorescence, as described in a previous study [28]. Samples were read in a dark room by two independent, experienced microscopists using a fluorescence microscope to establish the titer level. In cases of disagreement between the two microscopists, the sample was discarded and prepared again. Patients with detectable IgG antibodies in the screening sample had the remaining study samples further tested for antibodies against the corresponding microorganisms. The time of inclusion was chosen as baseline and we interpreted a fourfold rise in IgG antibodies at any of the follow-up visits (day 14, 3 months, 12 months) as serological evidence of recent exposure for all IFA assays. Patients with only weak antibody reactivity as indicated by a rise in antibody level from < 1:64 to 1:64 were excluded in the analyses. Further, a high IgG titer of ≥ 1:512 (clinical cut-off at SSI) or ≥ 1:128 (one titer above manufacturers recommendation) in at least one of the samples was considered evidence of prior exposure to SFG *Rickettsia* and *A*. *phagocytophilum*, respectively.

### ELISA

Day 14 and 3-month sera were tested for IgG antibodies against TBEV (187/188) and *Bbsl* (175/188), respectively, using ELISA.

For TBEV, the analysis was done using a commercially available IgG ELISA assay (Virion/Serion, Würzburg, Germany) at the Norwegian Institute of Public Health (NIPH), Oslo NO. We chose to analyze the day 14 samples for TBEV as the time from tick bite or EM was, at this point, expected to be sufficient for any seroconversion to have occurred. Equivocal results were retested using the same assay. Positive tests were reanalyzed with a different ELISA assay according to standard operating procedure (Euroimmun Anti-TBE Virus IgG, Lübeck, Germany).

The *Bbsl* analysis was performed at Sørlandet Hospital, Kristiansand NO, using a commercial ELISA kit (Enzygnost Borrelia, Lyme link VlsE/IgG), as described elsewhere [19]. At the time of inclusion, 50.0% of the patients (94/188) had detectable IgG antibodies against *Bbsl* [19] and to assess any further seroconversion, the 3-month sera were analyzed. For both *Bbsl* and TBEV, the IgG cut-off levels were set in accordance with the manufacturers’ recommendations.

### Clinical data

Data on concomitant symptoms was obtained through a patient diary kept for the first 14 days after inclusion [19]. The following 16 symptoms were inquired upon: tiredness, headache, arthralgia, neck stiffness, fever, palpitations, myalgia, sore throat, tender skin, dizziness, nausea, chest pain, diarrhea, chills, hot flushes, and coughing. Further, clinical data on the size, duration, diagnostic accuracy and appearance of the EM lesions were collected through clinical examination of the patients at days 0 and 14.

### Statistical methods

For comparing proportions, we used a chi-squared test (Χ^2^) or a Fisher’s Exact Test when the expected number in one or more cells in the crosstabs was < 5. Means were compared with a t-test and for the continuous variables we used a Mann–Whitney U test. Missing data are considered to be randomly distributed. *P*-values ≤ 0.05 were considered statistically significant. Analyses were performed using IBM SPSS Statistics for Windows (v. 25; IBM Corp., Armonk, NY, USA).

## Results

### Detection of microorganisms in biopsies and blood samples using molecular diagnostics

*Neoehrlichia mikurensis*, *Rickettsia* spp., *A*. *phagocytophilum* and *Babesia* spp*.* could not be detected in the skin biopsies (n = 139) using real-time PCR. Further, none of the above-mentioned microorganisms could be detected in the inclusion (day 0) serum samples of patients reporting fever (n = 8) or patients with an EM duration of ≥ 21 days (n = 69). For the latter, the amount of extractable samples was insufficient to perform analyses in 15.4% (29/188) of the patients. Additional real-time PCR analysis on the inclusion (day 0) samples of patients displaying at least a fourfold rise in anti-*Rickettsia* IgG compared to baseline revealed no detectable *Rickettsia* DNA.

### Serological analyses

An overview of the serological results is presented in Table 2. Four patients (cases 1–4; Table 2) displayed at least a fourfold rise in anti-*Rickettsia* IgG compared to baseline, one of whom displayed a concomitant rise in IgM antibodies (case 1; Table 2). 7/135 patients (5.2%) exhibited high anti-*Rickettsia* IgG titers of ≥ 1:512 in at least one of the samples (cases 5–11; Table 2).

**Table 2:** Overview of the EM patients displaying antibody reactivity against tick-borne microorganisms other than *Bbsl*

Case	Sex	Age	Microorganism and antibody class	Serology 1 at day 0 (baseline)	Serology 2 at day 14	Serology 3 after three months	Serology 4 after 12 months	*Bb. IgG 3 m*
**1**	F	67	SFGR IgG	** < 1:64**	NA	**1:128**	**1:128**	POS
			SFGR IgM	** < 1:64**		** < 1:64**	**1:256**	
			Ap IgG	1:64	NA	1:128	1:64	
			Ap IgM	< 1:20		< 1:20	< 1:20	
**2**	M	27	SFGR IgG	** < 1:64**	**1:128**	–	–	–
			SFGR IgM	< 1:64	< 1:64			
**3**	M	85	SFGR IgG	** < 1:64**	** < 1:64**	**1:128**	**1:128**	POS
			SFGR IgM	< 1:64	< 1:64	< 1:64	< 1:64	
**4**	F	50	SFGR IgG	** < 1:64**	** < 1:64**	**1:128**	** < 1:64**	POS
			SFGR IgM	< 1:64	< 1:64	< 1:64	< 1:64	
**5**	M	65	SFGR IgG	1:1024	1:1024	1:256	1:512	POS
			SFGR IgM	< 1:64	< 1:64	< 1:64	< 1:64	
**6**	F	59	SFGR IgG	1:512	1:1024	1:512	1:1024	NEG
			SFGR IgM	< 1:64	< 1:64	< 1:64	< 1:64	
			Ap IgG	1:128	1:128	1:128	1:64	
			Ap IgM	< 1:20	< 1:20	< 1:20	< 1:20	
**7**	F	49	SFGR IgG	1:512	1:512	1:512	1:1024	NEG
			SFGR IgM	< 1:64	< 1:64	< 1:64	< 1:64	
			Ap IgG	1:128	1:128	1:128	1:128	
			Ap IgM	< 1:20	< 1:20	< 1:20	< 1:20	
**8**	M	24	SFGR IgG	1:2048	1:2048	-	-	-
			SFGR IgM	< 1:64	< 1:64			
**9**	M	76	SFGR IgG	1:512	1:512	1:256	1:256	POS
			SFGR IgM	< 1:64	< 1:64	< 1:64	< 1:64	
**10**	F	51	SFGR IgG	1:4096	1:4096	1:4096	1:4096	POS
			SFGR IgM	< 1:64	< 1:64	< 1:64	< 1:64	
**11**	M	49	SFGR IgG	1:512	1:512	1:256	1:512	NEG
			SFGR IgM	< 1:64	< 1:64	< 1:64	< 1:64	
**12**	F	76	Ap IgG	1:64	1:64	1:128	1:64	POS
			Ap IgM	< 1:20	< 1:20	< 1:20	< 1:20	
**13**	F	60	Ap IgG	1:128	1:128	1:64	1:64	POS
			Ap IgM	< 1:20	< 1:20	< 1:20	< 1:20	
**14**	F	59	Ap IgG	1:64	1:128	1:128	1:128	POS
			Ap IgM	< 1:20	< 1:20	< 1:20	< 1:20	
**15**	M	55	Ap IgG	1:64	1:64	1:64	1:128	NEG
			Ap IgM	< 1:20	< 1:20	< 1:20	< 1:20	
**16**	M	50	Ap IgG	1:128	1:128	1:64	1:128	POS
			Ap IgM	1:40	1:40	< 1:20	20	

The titer values of the four patients displaying a fourfold rise in anti-*Rickettsia* IgG are printed in boldface

*SFGR* spotted fever group *Rickettsia*, *Ap* *Anaplasma phagocytophilum*

For *A*. *phagocytophilum*, no patients displayed a fourfold rise in IgG antibodies. However, 8/135 patients (5.9%) displayed increased levels of IgG at a titer of ≥ 1:128 in at least one of the samples (cases 12–16; Table 2). One of these patients had concomitant IgM antibodies against *A*. *phagocytophilum* (case 16; Table 2). Three patients exhibited antibody reactivity against both SFG *Rickettsia* and *A*. *phagocytophilum* (cases 1, 6 and 7; Table 2). No IgG antibodies could be detected against *B*. *microti*.

For TBEV, 9/187 patients (4.8%) displayed IgG antibodies on the day 14 visit. Six of these patients reported prior immunization against TBEV and one had previously been immunized against Yellow Fever, suggesting cross-reactivity. The two remaining patients had no prior history of flavivirus immunization suggesting possible prior exposure to TBEV or some other flavivirus. Only one (case 15; Table 2) of the nine patients with detectable IgG against TBEV had serological evidence of exposure to other tick-borne pathogens. The IgG antibodies were in this case vaccine-induced.

Analysis of the 3-month sera showed that 50.3% (88/175) of patients had detectable *Bbsl* IgG. For the 16 patients with serological evidence of co-infection, the proportion was 62.5% (10/16).

### Clinical data

An overview of the clinical data for the patients displaying serological evidence of exposure to SFG *Rickettsia* and *A*. *phagocytophilum* is presented in Table 3. No significant difference (p > 0.05) could be observed in the size, duration and appearance of the EM skin lesions or in the presence of concomitant symptoms between these patients and those *without* serological evidence of exposure to other tick-transmitted organisms than *Bbsl*. Among those *with* serological evidence of exposure to other tick-transmitted organisms than *Bbsl*, only one patient (case 14) reported fever. No fever or clinical signs of neuroinfection could be observed in the two patients displaying non-vaccine induced TBEV IgG during the study period.

**Table 3:** Clinical appearance of EM patients with or without exposition to other tick-borne agents

	EM with possible co-infection (n = 16)		EM without co-infection (n = 119)		p-value
	Statistics	n	Statistics	N	
Female, n (%)	8 (50%)	16	73 (61%)	118	0.36^e^
Age, mean (SD)	53.6 (20.7)	16	54.7 (15.3)	119	0.79^f^
Days from tick-bite to EM, median (IQR)	21 (8–25)	11	11 (5–21)	74	0.21^g^
Days from EM to treatment, median (IQR)	5 (2–20)	15	7 (2–16)	103	0.87^g^
Diagnostic accuracy “very sure”^a^, n (%)	12 (75%)	16	81 (68%)	119	0.78^h^
EM duration, median (IQR)	14 (8–28)	16	14 (9–21)	119	0.66^g^
Concomitant symptoms^b^, median (IQR)	2 (0–3)	16	1 (0–2)	119	0.50^g^
Fever, n (%)	1 (6.3%)	16	4 (3.4%)	117	0.48^h^
EM color^c^, mean (SD)	3.6 (0.89)	16	3.27 (0.89)	119	0.13^f^
EM diameter (cm), median (IQR)	10 (6–11)	16	10 (7–14)	116	0.69^g^
EM appearance “Bulls eye”, n (%)	11 (69%)	16	59 (50%)	117	0.16^e^
*Bbsl* biopsy pos., n (%)	12 (86%)	14	58 (66%)	88	0.22^h^
Antibiotic treatment^d^, n (%)	A: 2 (13%) B: 5 (31%) C: 9 (56%)	16	A: 38 (32%) B: 41 (34%) C: 40 (34%)	119	N/A

*IQR*  interquartile range, *SD*  standard deviation

^a^Diagnostic accuracy scored by including GP, “not sure”, “sure” or “very sure”

^b^Number of the 16 symptoms registered: Tiredness, headache, arthralgia, neck stiffness, fever, palpitations, myalgia, sore throat, tender skin, dizziness, nausea, chest pain, diarrhea, chills, hot flushes, coughing

^c^Colour of the EM at the day of inclusion, grade 1–5 (light red—dark red).

^d^A = phenoxymethylpenicillin, B = Amoxicillin. C = Doxycycline. Treatments were randomized in the trial [19].

^e^X^2^-test

^f^t-test

^g^Mann–Whitney U test

^h^Fisher’s Exact Test

## Discussion

We found (1) no molecular evidence of other tick-borne microorganisms than *Bbsl* being present in the skin lesions of clinically diagnosed EM patients in Norwegian general practice and (2) a generally low proportion of these patients displaying serological evidence of exposure to the studied microorganisms. Of those displaying serological evidence of exposure, only four exhibited a significant rise in IgG antibodies between the initial visit and follow-up, suggesting recent exposure.

These findings are in line with a previous Dutch study which examined 291 EM patients and detected DNA from other tick-borne microorganisms than *Bbsl* in the EDTA-blood of only 8 (3%) using real-time PCR [13]. Given that PCR analysis of blood has been reported to have a lower sensitivity than serological analysis for several tick-borne pathogens [25–27], it is possible that this was an underreporting of the true prevalence. A more recent study, combining serological and molecular methods of detection, also showed low rates of concomitant exposure to more than one tick-borne microorganism in EM patients diagnosed in the Nordic countries [11]. This latter study was, however, limited by a small study population, short follow-up and no examination of skin biopsies.

Strengths of our study include the use of both molecular and serological methods of detection, recruitment of patients with a clinical diagnosis of EM in a general practice setting, a large proportion of PCR-verified EM skin lesions ascertaining a previous tick-bite and the use of both serum samples and skin biopsies for the analyses. Of the 105 patients who were analyzed with both PCR and serology, 70 (67%) had detectable *Bbsl* DNA in their skin biopsies [19], thus confirming a diagnosis of EM. 14 of the 16 patients with serological evidence of other tick-borne infections had their skin biopsies analyzed for *Bbsl* in the original trial [19], with detectable *Bbsl* DNA being found in 12 (86%) of these biopsies, again confirming a high percentage of true EM lesions among the studied patients. The use of *R*. *ricketsii* as antigen could have reduced the sensitivity of our serological assay for other species of SFG *Rickettsia*. However, considering the general homology among species of SFG *Rickettsia*, we expect it to be capable of detecting other species of SFG *Rickettsia* as well, as has been suggested in a previous study [29]. Further, no serological assay for the detection of *N*. *mikurensis* was available at the time of this study. However, antibodies against *A*. *phagocytophilum* have been shown to cross-react with *N*. *mikurensis* [30] and the detected antibodies against *A*. *phagocytophilum* could thus represent exposure to *N*. *mikurensis*. Moreover, serum samples, and not whole blood, were used for parts of the PCR analyses which could have reduced the clinical sensitivity for some tick-transmitted microorganisms such as the intraerythrocytic *Babesia* spp. Additional TBE testing in samples collected at three months might have revealed some additional seroconversions in patients with a longer than average incubation period or delayed humoral immune response. However, as the study was based on samples collected through a previous study [19], we used the patient material that was available to our disposal.

This study indicates that co-infections are rare in Norwegian patients with EM. Antibodies against SFG *Rickettsia* and *A*. *phagocytophilum* were detected in some of these patients. However, no significant difference in clinical symptoms, appearance, or duration of EM skin lesions in patients with or without antibodies against SFG *Rickettsia* and *A*. *phagocytophilum,* was detected. However, as only four of the patients displayed a fourfold rise in titer, it is impossible to tell if most of the detected antibodies reflect recent exposure or prior infection unrelated to the current symptoms. A median duration of 21 days elapsed from the time of the tick-bite until the EM was noticed (Table 3) and it is likely that seroconversion occurred during this time frame for some of the patients. As infections with *N*. *mikurensis* have primarily been reported in chronically ill immunosuppressed patients [31–33], it is also important to emphasize that the participants of our study reported baseline general health at a comparable level to the background population and that active treatment with immunosuppressants was an exclusion criteria for the original trial [19].

EM is a clinical diagnosis and in the Nordic countries the recommended treatment for EM without fever or other signs of disseminated disease is phenoxymethylpenicillin [34, 35]. Our study does not support a change in practice regarding this first-line treatment. Of note, 44% of the participants displaying serological evidence of exposure to *Rickettsia* or *Anaplasma* received treatment with phenoxymethylpenicillin or amoxicillin (Table 3), antibiotics with no effect on obligate intracellular organisms.

## Conclusions

Our study indicates that co-infections with *N*. *mikurensis*, *A*. *phagocytophilum*, SFG *Rickettsia*, *Babesia* spp. and TBEV are uncommon in Norwegian patients with EM. Despite detecting antibodies against SFG *Rickettsia* and *A*. *phagocytophilum* in some of these patients, no clinical implications could be demonstrated, and the clinical significance of these findings remains unknown.

## Data Availability

The data for analyses during the current study are available from the corresponding author on reasonable request.

## Abbreviations

*Bbsl*: *Borrelia burgdorferi* Sensu lato
BHQ: Black Hole Quencher
ELISA: Enzyme-Linked ImmunoSorbent Assay
EM: Erythema migrans
FAM: 6-Carboxy-fluorescine
IFA: Indirect immunofluorescence antibody
IQR: Interquartile range
LB: Lyme borreliosis
MGB: Minor groove binder
PCR: Polymerase chain reaction
SD: Standard deviation
SFG(R): Spotted fever group (*Rickettsia*)
TBEV: Tick borne encephalitis virus

## References

[1] Stanek G, Wormser GP, Gray J, Strle F (2012). Lyme borreliosis. Lancet Lond Engl.

[2] Quarsten H, Skarpaas T, Fajs L, Noraas S, Kjelland V (2015). Tick-borne bacteria in *Ixodes ricinus* collected in southern Norway evaluated by a commercial kit and established real-time PCR protocols. Ticks Tick-Borne Dis.

[3] Jenkins A, Kristiansen BE, Allum AG, Aakre RK, Strand L, Kleveland EJ (2001). *Borrelia burgdorferi* sensu lato and *Ehrlichia* spp. in Ixodes ticks from southern Norway. J Clin Microbiol.

[4] Larsson C, Hvidsten D, Stuen S, Henningsson AJ, Wilhelmsson P (2018). “Candidatus *Neoehrlichia mikurensis*” in *Ixodes ricinus* ticks collected near the Arctic Circle in Norway. Parasit Vectors.

[5] Mysterud A, Easterday WR, Qviller L, Viljugrein H, Ytrehus B (2013). Spatial and seasonal variation in the prevalence of *Anaplasma phagocytophilum* and *Borrelia burgdorferi* sensu lato in questing *Ixodes ricinus* ticks in Norway. Parasit Vectors.

[6] Henningsson AJ, Hvidsten D, Kristiansen B-E, Matussek A, Stuen S, Jenkins A (2015). Detection of *Anaplasma phagocytophilum* in *Ixodes ricinus* ticks from Norway using a realtime PCR assay targeting the Anaplasma citrate synthase gene gltA. BMC Microbiol.

[7] Wilhelmsson P, Lövmar M, Krogfelt KA, Nielsen HV, Forsberg P, Lindgren PE (2020). Clinical/serological outcome in humans bitten by Babesia species positive *Ixodes ricinus* ticks in Sweden and on the Åland Islands. Ticks Tick-Borne Dis..

[8] Koetsveld J, Tijsse-Klasen E, Herremans T, Hovius JWR, Sprong H (2016). Serological and molecular evidence for spotted fever group Rickettsia and *Borrelia burgdorferi* sensu lato co-infections in The Netherlands. Ticks Tick-Borne Dis.

[9] Lindblom A, Wallménius K, Nordberg M, Forsberg P, Eliasson I, Påhlson C (2013). Seroreactivity for spotted fever rickettsiae and co-infections with other tick-borne agents among habitants in central and southern Sweden. Eur J Clin Microbiol Infect Dis.

[10] Tijsse-Klasen E, Sprong H, Pandak N (2013). Co-infection of *Borrelia burgdorferi* sensu lato and *Rickettsia* species in ticks and in an erythema migrans patient. Parasit Vectors.

[11] Ocias LF, Wilhelmsson P, Sjöwall J, Henningsson AJ, Nordberg M, Jørgensen CS (2020). Emerging tick-borne pathogens in the Nordic countries: a clinical and laboratory follow-up study of high-risk tick-bitten individuals. Ticks Tick-Borne Dis..

[12] Grankvist A, Sandelin LL, Andersson J, Fryland L, Wilhelmsson P, Lindgren P-E (2015). Infections with Candidatus *Neoehrlichia mikurensis* and cytokine responses in 2 persons bitten by ticks, Sweden. Emerg Infect Dis.

[13] Jahfari S, Hofhuis A, Fonville M, van der Giessen J, van Pelt W, Sprong H (2016). Molecular detection of tick-borne pathogens in humans with tick bites and erythema migrans, in the Netherlands. PLoS Negl Trop Dis.

[14] Quarsten H, Grankvist A, Høyvoll L, Myre IB, Skarpaas T, Kjelland V (2017). Candidatus *Neoehrlichia mikurensis* and *Borrelia burgdorferi* sensu lato detected in the blood of Norwegian patients with erythema migrans. Ticks Tick-Borne Dis.

[15] Porskrog A, Himmelstrup B, Wennerås C. Tick-borne Candidatus *Neoehrlichia mikurensis* detected by 16S rRNA PCR on a skin punch biopsy—first case in Denmark (poster number P0130). European Congress of Clinical Microbiology and Infectious Diseases (ECCMID) 2015. In Copenhagen, Denmark.

[16] Matei IA, Estrada-Peña A, Cutler SJ, Vayssier-Taussat M, Varela-Castro L, Potkonjak A (2019). A review on the eco-epidemiology and clinical management of human granulocytic anaplasmosis and its agent in Europe. Parasit Vectors.

[17] Parola P, Paddock CD, Raoult D (2005). Tick-borne rickettsioses around the world: emerging diseases challenging old concepts. Clin Microbiol Rev.

[18] Nilsson K (2009). Septicaemia with *Rickettsia helvetica* in a patient with acute febrile illness, rash and myasthenia. J Infect.

[19] Eliassen KE, Reiso H, Berild D, Lindbæk M (2018). Comparison of phenoxymethylpenicillin, amoxicillin, and doxycycline for erythema migrans in general practice. A randomized controlled trial with a 1-year follow-up. Clin Microbiol Infect.

[20] Wilhelmsson P, Lindblom P, Fryland L, Ernerudh J, Forsberg P, Lindgren P-E (2013). Prevalence, diversity, and load of Borrelia species in ticks that have fed on humans in regions of Sweden and Åland Islands, Finland with different Lyme borreliosis incidences. PLoS ONE.

[21] Stensvold CR, Al Marai D, Andersen LO, Krogfelt KA, Jensen JS, Larsen KS (2015). *Babesia* spp. and other pathogens in ticks recovered from domestic dogs in Denmark. Parasit Vectors.

[22] Stenos J, Graves SR, Unsworth NB (2005). A highly sensitive and specific real-time PCR assay for the detection of spotted fever and typhus group Rickettsiae. Am J Trop Med Hyg.

[23] Labbé Sandelin L, Tolf C, Larsson S, Wilhelmsson P, Salaneck E, Jaenson TGT (2015). Candidatus *Neoehrlichia mikurensis* in ticks from migrating birds in Sweden. PLoS ONE.

[24] Andersson MO, Víchová B, Tolf C, Krzyzanowska S, Waldenström J, Karlsson ME (2017). Co-infection with Babesia divergens and *Anaplasma phagocytophilum* in cattle (Bos taurus), Sweden. Ticks Tick-Borne Dis.

[25] Hansmann Y, Jaulhac B, Kieffer P, Martinot M, Wurtz E, Dukic R (2019). Value of PCR, serology, and blood smears for human granulocytic Anaplasmosis diagnosis, France. Emerg Infect Dis.

[26] Paris DH, Dumler JS (2016). State of the art of diagnosis of rickettsial diseases: the use of blood specimens for diagnosis of scrub typhus, spotted fever group rickettsiosis, and murine typhus. Curr Opin Infect Dis.

[27] Krause PJ, Telford SR, Ryan R, Conrad PA, Wilson M, Thomford JW (1994). Diagnosis of babesiosis: evaluation of a serologic test for the detection of *Babesia microti* antibody. J Infect Dis.

[28] Kantsø B, Svendsen CB, Jørgensen CS, Krogfelt KA (2009). Evaluation of serological tests for the diagnosis of rickettsiosis in Denmark. J Microbiol Methods.

[29] Nilsson K, Lindquist O, Påhlson C (1999). Association of *Rickettsia helvetica* with chronic perimyocarditis in sudden cardiac death. Lancet Lond Engl.

[30] Wass L, Grankvist A, Mattsson M, Gustafsson H, Krogfelt K, Olsen B (2018). Serological reactivity to *Anaplasma phagocytophilum* in neoehrlichiosis patients. Eur J Clin Microbiol Infect Dis.

[31] Welinder-Olsson C, Kjellin E, Vaht K, Jacobsson S, Wennerås C (2010). First case of human “Candidatus *Neoehrlichia mikurensis*” infection in a febrile patient with chronic lymphocytic leukemia. J Clin Microbiol.

[32] Grankvist A, Andersson P-O, Mattsson M, Sender M, Vaht K, Höper L (2014). Infections with the tick-borne bacterium “Candidatus *Neoehrlichia mikurensis*” mimic noninfectious conditions in patients with B cell malignancies or autoimmune diseases. Clin Infect Dis.

[33] Wennerås C (2015). Infections with the tick-borne bacterium Candidatus *Neoehrlichia mikurensis*. Clin Microbiol Infect.

[34] Reiso H, Berild D. Antibiotikabruk i primærhelsetjenesten: http://www.antibiotikaiallmennpraksis.no/index.php?action=showtopic&topic=Zf9ZUXe2. 2016.

[35] Ocias LF, Jensen BB, Knudtzen FC, Skarphedinsson S, Dessau RB. Clinical manifestations, diagnosis and treatment of Lyme borreliosis. Ugeskr Laeger. 2017;179(18) (in Danish). https://ugeskriftet.dk/videnskab/klinik-diagnostik-og-behandling-af-lyme-borreliose. 28473022

